# Multimodal evaluation of an interphotoreceptor retinoid-binding protein-induced mouse model of experimental autoimmune uveitis

**DOI:** 10.1038/s12276-022-00733-z

**Published:** 2022-03-09

**Authors:** Jee Myung Yang, KyungA Yun, Jehwi Jeon, Hae Young Yang, Bora Kim, Sunhong Jeong, Junyeop Lee, Wang-Yuhl Oh, Akiyoshi Uemura, Joon Seon Song, Pilhan Kim, Joo Yong Lee

**Affiliations:** 1 Department of Ophthalmology, Asan Medical Center, University of Ulsan College of Medicine, 88 Olympic-ro 43-gil, Songpa-gu, Seoul 05505 Republic of Korea; 2 Graduate School of Medical Science and Engineering, Korea Advanced Institute of Science and Technology, Daejeon, Korea; 3 Graduate School of Mechanical Engineering, Korea Advanced Institute of Science and Technology, Daejeon, Korea; 4 Department of Retinal Vascular Biology, Nagoya City University Graduate School of Medical Sciences, Nagoya, Japan; 5 Department of Pathology, Asan Medical Center, University of Ulsan College of Medicine, 88 Olympic-ro 43-gil, Songpa-gu, Seoul 05505 Republic of Korea

**Keywords:** Vasculitis, Imaging, Retina, Retina

## Abstract

We aimed to characterize the vascular phenotypes of an experimental autoimmune retinal uveitis (EAU) model induced by interphotoreceptor retinoid-binding protein (IRBP) using multimodal imaging techniques. We systemically administered IRBP or vehicle to adult C57BL/6 mice. Fundus photography, optical coherence tomography (OCT), in vivo live confocal imaging using different tracers, OCT angiography (OCTA), and electroretinography (ERG) were performed after IRBP immunization. Hematoxylin and eosin and immunofluorescence staining were performed to characterize the immune response and vascular permeability. Mice with EAU exhibited perivascular inflammation, vitritis, and superficial retinal inflammation on fundus photography and OCT. H&E revealed immune cell infiltration in the perivascular area of the retina and choroid accompanied by a significant degree of perivasculitis that subsequently damaged photoreceptors 3 weeks postimmunization. Immunofluorescence staining showed subsequent transcytosis induction after local microglial activation followed by neutrophil recruitment in the perivascular area. Transcytosis in the superficial and deep vascular areas was improved by immune cell suppression. Intravital in vivo confocal imaging showed signs of neutrophil infiltration and obstructive vasculitis with perivascular leakage 3 weeks postimmunization. OCTA revealed a significant decrease in vascular flow in the deep capillary layer of the retina. Functional analysis showed that scotopic responses were intact at 2 weeks; however, normal photopic and scotopic responses were hardly detected in mice with EAU mice at 3 weeks postimmunization. Our data suggest that inflammatory cell activation and subsequent transcytosis induction in endothelial cells might be a major pathogenic factor for vascular leakage in uveitis, providing new insights into the pathophysiology of retinal vasculitis in noninfectious uveitis.

## Eye disease: Understanding autoimmune eye inflammation

Studying a mouse model of autoimmune uveitis, a damaging form of eye inflammation affecting the retina and choroid of the eye, reveals new cellular and molecular details of how blood vessel inflammation can damage the retina. Researchers in South Korea and Japan led by Joo Yong Lee at the University of Ulsan, Seoul, initiated autoimmune uveitis in mice by administering retinoid-binding protein, which is known to stimulate autoimmune changes which model aspects of the human disease. Their work revealed that the inflammation caused by the autoimmune response makes the blood vessels supplying the retina more permeable to a variety of large molecules. This increased permeability, due to a membrane transport process called transcytosis, was preceded by specific cellular changes. This deeper understanding of the pathology of uveitis could help research towards new treatments.

## Introduction

Uveitis is a spectrum of diseases characterized by uveal inflammation that leads to 5–10% visual loss^1^. Autoimmune or noninfectious uveitis, which involves systemic inflammatory conditions, is an intractable and vision-threatening condition of the neuroretina^2^. It involves inflammation of the retinal vessels, leading to vascular leakage and occlusion that can result in significant destruction of the nonregenerating cells of the retina^3^. Although the need to understand and control vasculitis in patients with uveitis is increasing, literature on vascular environmental changes in the uveitic retina is scarce.

Patients with noninfectious uveitis have aberrant systemic immunity and often show autoimmune responses to retinal proteins, such as arrestin and interphotoreceptor retinoid-binding protein (IRBP)^4^. IRBP, which is an essential part of the visual cycle in photoreceptor cells^5^, is widely used as an autoantigen to induce experimental autoimmune uveitis (EAU)^3,6^. EAU induced by IRBP immunization is characterized by vascular leakage and edema; however, how the inner blood–retinal barrier (BRB) is damaged has not been thoroughly studied^3^. Similar to the blood–brain barrier (BBB), the BRB is largely dependent on (1) tight junctions (paracellular transport restriction) and (2) inactive transcytosis (transcellular transport restriction)^7,8^. Although the formation and disruption of tight junctions has been increasingly characterized, the regulatory mechanisms of transcytosis remain poorly elucidated. As recent cutting-edge studies have focused on the role of transcytosis in pathologic leakage in central nervous system (CNS) vessels^9–13^, emerging evidence indicates a role for increased transcytosis in the pathogenesis of major retinal vascular diseases^14–17^.

With the rapid development of retinal imaging, it is possible to obtain images of the deeper retinal layer with higher contrast and resolution^18^. Previously, we developed experimental tools to visualize retinal vessels in vivo for live confocal imaging and OCTA for deep retinal vessel imaging^19,20^. Therefore, by utilizing these technologies, we have obtained multimodal images of retinal vessels in EAU mice. In the present study, we show that neutrophils are recruited, vascular flow is decreased, microglia are activated, and transcytosis is induced in the EAU mouse retina and suggest that inflammatory cell activation might precede and have a role in increasing vascular permeability by transcytosis induction. To our knowledge, this is the first study to demonstrate that the IRBP-induced EAU model exhibits inflammation-induced vascular hyperpermeability resulting from transcytosis induction and suggest a pathologic role for transcytosis in human retinal vasculitis.

## Materials and methods

### Mice

All experimental mice were on the C75BL/6J background and housed in a pathogen-free animal facility at the Asan Institute for Life Sciences. The mice were anesthetized by intraperitoneal injection of a mixture of tiletamine hydrochloride, zolazepam hydrochloride (Zoletil, 0.4 mL/kg; Virbac Laboratories, Carros, France) and xylazine (Rompun, 0.6 mL/kg; Bayer Korea Ltd., Seoul, Korea). All animal care and experimental procedures were performed according to a protocol that was approved by the Institutional Animal Care Committee (approval number 2019-14-173) and was in accordance with the Association for Research in Vision and Ophthalmology statement for the use of animals in ophthalmic and vision research. *LysM-GFP* mice were generously provided by Professor M. Kim (University of Rochester, Rochester, NY, USA). *Cx3cr1-GFP* mice, in which enhanced green fluorescent protein (eGFP) is expressed on microglia, monocytes, and dendritic cells, were purchased from Jackson Laboratory (stock no. 005582; Bar Harbor, ME, USA).

### EAU model

**Fig. 1:**
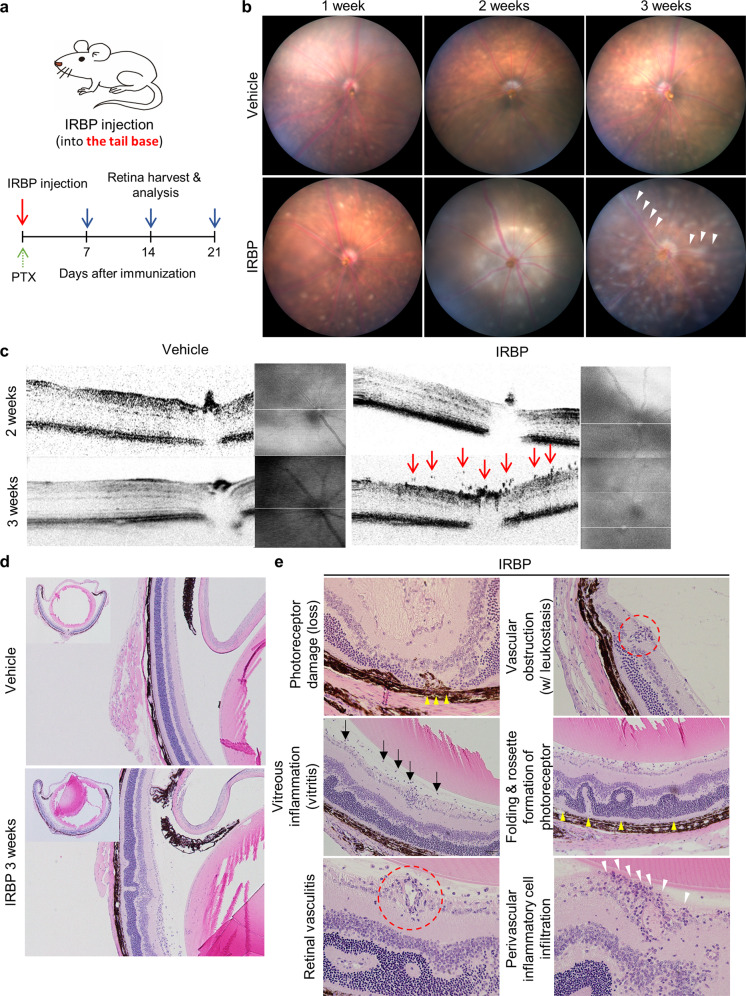
Interphotoreceptor retinoid-binding protein (IRBP)-immunized mice present perivascular inflammation. **a** Schematic diagram of the experimental schedule used to contract the IRBP-induced experimental autoimmune uveitis (EAU) model. IRBP peptide emulsified in complete Freund’s adjuvant (CFA) was injected into the tail base, and adjuvant (purified *Bordetella pertussis* toxin) was administered intraperitoneally. **b** Fundus images of mice with EAU showing diffuse perivascular inflammation evidenced by vascular sheathing (white arrowhead). **c**. Optical coherence tomography (OCT) images of mice with EAU showing inflammatory cells on the preretinal surface and in the superficial retinal layers (red arrows). **d**, **e** Histologic features indicating retinal vasculitis in IRBP-immunized mice. **d** Hematoxylin and eosin staining of the eyecup and retinal layer in the EAU mouse model. **e** Representative images showing various signs of vascular inflammation and subsequent retinal damage in mice with EAU. The arrow, arrowheads, and red-dotted circles indicate areas representative of the different phenotypes.

To induce inflammatory transcytosis of the retinal and choroidal vessels, a mouse model of EAU was established by immunization with IRBP 1-20 (GenScript, Piscataway, NJ, USA) as previously described^21^. Briefly, IRBP was emulsified in complete Freund’s adjuvant (CFA, 1:1 vol/vol; Sigma-Aldrich, St. Louis, MO, USA) and systemically administered intramuscularly (0.2 mL emulsification per mouse, at the tail base) (Fig. 1a). Purified *Bordetella pertussis* toxin (PTX) was administered intraperitoneally as an adjuvant (1.5 μg per mouse). Examinations were performed on the indicated days for analysis.

### Neutrophil and microglia suppression model

To suppress neutrophil infiltration, a high dose (10 mg/kg) of Ly6G+ monoclonal antibody (stock no. 551459; BD Biosciences, Franklin Lakes, NJ, USA) was intraperitoneally injected into *LysM-GFP* neutrophil reporter mice at 3-day intervals (Fig. 4A)^22^. Additionally, to suppress microglial activation, minocycline (45 ml/kg for induction, 22.5 ml/kg for booster; M9511-100MG, Sigma-Aldrich) was intraperitoneally injected into *Cx3cr1-GFP* microglial reporter mice at 3-day intervals (Fig. 4D)^23^.

### Fundus photography and OCT

For intravital retinal imaging, the pupils were dilated with Mydrin-P (0.5% tropicamide and 0.5% phenylephrine hydrochloride; Santen Pharmaceuticals, Osaka, Japan) after anesthesia. A physiological solution (Hycell solution, 2% hydroxypropylmethylcellulose; Samil Pharmaceuticals, Korea) was placed on the cornea, and a microscope coverslip was used to equalize refraction. ﻿The retinas were observed for clinical signs of uveitis using fundus imaging and OCT (OCT; IIS Science)^24^.

### Intravital retinal imaging using custom-built high-speed laser-scanning retinal confocal microscopy

In vivo retinal imaging was performed using a custom-built laser-scanning confocal microscopy system modified for retinal imaging from a previously developed intravital confocal imaging platform^19^. Three continuous-wave laser sources, i.e., a 488-nm diode laser module [Cobolt MLD 488 (HÜBNER Photonics, San Jose, CA, USA), 561-nm DPSS laser (Cobolt Jive; HÜBNER Photonics), and 640-nm diode laser module (Cobolt MLD 640; HÜBNER Photonics), were used as excitation light sources. A raster scanning pattern of the excitation laser was generated using a scanner system comprising a rotating polygonal mirror (MC-5; Lincoln Laser, Phoenix, AZ, USA) and galvanometer-based scanning mirror (6230H; Cambridge Technology, Bedford, MA, USA) and then delivered to the back aperture of an imaging lens. A high NA objective lens (PlanApoλ, numerical aperture = 0.75; Nikon, Tokyo, Japan) was used as the imaging lens to provide a wide-angle fluorescence image of the retina. Fluorescence emissions were detected using a multialkali photocathode photomultiplier tube (R9110; Hamamatsu Photonics, Hamamatsu, Shizuoka, Japan). The electric signal was digitized using a frame grabber (Solios; Matrox, Dorval, Quebec, Canada) and reconstructed to images with a size of 512 × 512 pixels per frame in real time. The anesthetized mice were placed on an articulating base ball stage (SL20; Thorlabs, Newton, NJ, USA) fixed to the XYZ translation stage (3DMS; Sutter Instruments, Novato, CA, USA).

For in vivo imaging, the mice were anesthetized with a mixture of Zoletil (30 mg/kg) and xylazine (10 mg/kg) via intramuscular injection. The body temperature of the anesthetized mice was maintained at 36 °C using a homeothermic temperature monitoring and control system (RightTemp; Kent Scientific, Torrington, CT, USA) to prevent the abrupt formation of cold cataracts, which hampers imaging of the retina. Yohimbine (2 mg/kg), an antagonist of xylazine, was injected to facilitate postanesthesia recovery and stabilization of the cardiovascular system. Ophthalmic ointment and artificial tears were applied to avoid corneal injury and dryness. To visualize retinal vascular obstruction and leakage, 50 mg/kg FITC-dextran (fluorescein isothiocyanate/dextran 2 M, stock no. FD2000S; Sigma-Aldrich) and 100 mg/kg TRITC-dextran (tetramethylrhodamine isothiocyanate/dextran 155k, T1287, 4.4k T1037; Sigma-Aldrich) were injected via the tail vein. An anti-Ly6 g antibody (25 mg; BD Biosciences) conjugated to the far-red fluorophore Alexa Fluor 647 (stock no. A20006; Invitrogen, Waltham, MA, USA) was intravenously injected to fluorescently label monocytes and neutrophils to visualize sequestration and infiltration.

Vascular and optic nerve head leakages were assessed by quantifying the amount of TRITC-dextran outside of the vascular and optic nerve borders (in the absence of CD31). Vascular occlusion was also defined as the CD31-positive/dextran-negative area in the vessels. Disconnected vessels were defined as branches of retinal vessels without capillary branches. The data were processed using “surface function” in IMARIS, and an automatic filter below 10 voxels and 22.5–30.0 absolute intensity were used to acquire qualified data to remove noise and artifacts.

### OCTA

The mouse eyes were imaged using a prototype OCTA system^20,25^. A commercial wavelength-swept laser (Axsun Technologies, Billerica, MA, USA) with a center wavelength of 1050 nm and a repetition rate of 200 kHz was used as a light source. The incident optical power on the cornea was approximately 1.4 mW. The axial and transverse resolutions on the retina were 7.6 and 11.1 μm, respectively. Each B-scan location was scanned three times using interscan times of 1.6, 1.92, 2.56, 3.84, and 5.12 ms, which correspond to 320, 384, 512, 768, and 1024 A-lines per B-scan, respectively. A three-dimensional volume consisted of 768 B-scans acquired in 34.5 sec. OCT angiograms were generated by calculating normalized complex differential variance. For *en face* projection of the different vascular plexuses, the retinal and choroidal layers were segmented semiautomatically. The retinal layer was further segmented into the superficial capillary plexus (SCP) (from the retinal nerve fiber layer to the inner plexiform layer) and the deep capillary plexus (DCP) (from the inner nuclear layer to the outer plexiform layer). For each layer, a 2D *en face* angiogram was obtained by maximum projection of the 3D volumetric angiogram, and the BFI was calculated as the sum of all pixel values above a threshold value divided by the number of corresponding pixels for semiquantitative blood flow analysis.

### ERG

ERG was performed as described previously^20,24^. The mice were adapted to the dark for at least 12 h before recording. Only dim red light was used during the measurement of scotopic and photopic responses. All ERG recordings were performed using a Phoenix Micron IV system (Phoenix Research Labs, Pleasanton, CA, USA) and analyzed using LabScribeERG software (Version 3; Phoenix Research Labs). After anesthesia, the pupils were dilated, and the cornea was located using a gold-plated objective lens. Needle electrodes were placed at the forehead (reference) and tail (ground) for measurement. Infrared light was used to maintain body temperature after recording until the animals regained consciousness.

### Histology

For H&E staining, eyes were fixed overnight in 4% paraformaldehyde (PFA), embedded in paraffin, and cut into 4-µm sections. To grade the clinical severity of uveitis according to photoreceptor damage, inflammatory changes, and vasculitis, H&E-stained images were used for histopathologic evaluation as described previously^26^. Grading was performed independently by two researchers (JMY, JYL) in a blinded fashion. For whole-mount retinas, the eyes were enucleated and immediately fixed in 4% PFA in phosphate-buffered saline (PBS) for 20 min at room temperature (RT), and the retinas were isolated. The isolated retinas were subsequently fixed in 1% PFA for 1 h at RT. For cryosectioned retinas, the eyes were enucleated and snap-frozen in OCT compound after overnight fixation in 4% PFA and gradient dehydration in sucrose. The processed retinas were blocked with 5% normal goat or donkey serum in PBST (0.5% Triton X-100 in PBS) and incubated overnight at 4 °C with the following primary antibodies: anti-PECAM (clone 2H8; hamster, Millipore, MAB1398Z), anti-IBA1 (rabbit, WAKO, 019-19741), anti-PLVAP (rat, BD Biosciences, 553849), anti-Ly6G (rat, Abcam. RB6-8C5), and Cy3-conjugated anti-IgG (mouse, Sigma-Aldrich). After washing in 0.5% PBST, the samples were incubated for 4 h at RT with Alexa Fluor-conjugated species-specific secondary antibodies. For nuclear staining, the samples were incubated with DAPI/Hoechst. Subsequently, the samples were washed in 0.5% PBST for a sufficient amount of time and flat-mounted in mounting medium (Vectashield, Vector Laboratories, Burlingame, CA, USA). Immunofluorescence images were acquired using a Zeiss LSM 800 confocal microscope (Carl Zeiss, Berlin, Germany).

### Morphometric analysis

All quantifications were performed using high-resolution images collected with a Zeiss confocal microscope (Carl Zeiss)^14^. ImageJ software (http://rsb.info.nih.gov/ij) or Zen 2012 software (Carl Zeiss) was used for morphometric analyses. The retinal vascular density was calculated as a percentage by dividing the CD31+ retinal vessel area by the total area of interest. The numbers of retinal microglia and neutrophils were determined by counting IBA1+ cells and Ly6G+ cells, respectively, in a minimum of four randomly selected 420 × 420 μm fields per sample. Microglia were considered activated when they showed one of the following features: retraction of their processes, loss of their “tile span” distribution, an increased population, or an amoeboid shape. To quantify activated microglia, individual retinal microglia were automatically identified as those with a soma size greater than 22.5 μm by using the “Spot” function in IMARIS commercial image analysis software (Bitplane, Belfast, UK). activated “amoeboid” microglia were defined as microglia with a soma size greater than 50 μm and dendrite size less than 50 μm^27^. The staining intensities of specific molecules were analyzed in at least four representative areas of the retinal vessels or other indicated vascular region per retina and then averaged. The values were normalized to the background signals in nonvascularized areas, and their ratios were compared to those of controls; the values are indicated as fold changes or percentages.

### Statistics

Statistical analyses were performed using R version 3.6.2 (R Foundation for Statistical Computing, Vienna, Austria) and GraphPad Prism v.7.0 (GraphPad Software, San Diego, CA, USA). The data are presented as the mean ± standard deviation (SD). The Shapiro–Wilk test was used to assess the normality of the data. The Mann–Whitney *U* test was used to compare continuous nonnormally distributed variables between two groups, and Student’s *t* test was used to compare normally distributed variables. Multiple comparisons among groups were determined by one-way ANOVA followed by Dunnett’s post-hoc test. A *P* value <0.05 was considered statistically significant unless otherwise indicated.

## Results

### Mice with EAU exhibited retinal vasculitis

In this study, we used human IRBP peptides 1–20, which resemble the bovine and murine IRBP sequences^21^. Figure 1a shows a schematic diagram of the experimental schedule of IRBP immunization. IRBP was systemically administered via the intramuscular route (tail base). After immunization with IRBP, mice with EAU showed a gradual increase in perivascular inflammation, which was significant at 3 weeks postimmunization (Fig. 1b). Optical coherence tomography (OCT) images showed inflammatory cells on the preretinal surface and in the superficial retinal layers at 3 weeks postimmunization (Fig. 1c). These results were comparable to those of previous studies demonstrating that inflammation peaks 3 weeks after IRBP immunization^22,28^.

Next, to examine the inflammatory status and changes in retinal structure, hematoxylin and eosin (H&E)-stained images of retinal cross-sections of mice with EAU were analyzed (Fig. 1d, e). Notably, a significant number of inflammatory cells infiltrated the peri- and intravascular areas in mice with EAU (Table 1). These infiltrated inflammatory cells induced leukostasis, leading to vascular obstruction. As a consequence of posterior segment inflammation, photoreceptor damage and retinal folds were observed, leading to a significant decline in visual function.

**Table 1:** Histopathologic grading of the retinas of mice with interphotoreceptor retinoid-binding protein (IRBP)-induced EAU at 3 weeks.

Grade	Photoreceptor damage			Infiltration								Retinal vasculitis		
	Folds	Loss	Detachment	Choroid			Retina			Neo vascularization	Vitreous	% of vessels affected	Peri-vasculitis	Thrombi
				Non granulomatous	Granuloma		Non granulomatous	Granuloma						
					No. section	Size (units)		No. section	Size (units)					
0.5				+			+							
1	+		+	+	1	0.2	+	1	0.2		+		+	
2	+	+	+	+	1~2	1	+	1~2	0.5		+	10%	+	+
3	+	++	++	++	2~3	3	+	3	1	+	++	20–50%	+	+
4	+	++	+++	+++	>3	å 5	++	>3	>1	+	+++	>50%	+	+

Phenotype frequency: +, 1–25%; ++, 25–50%; +++, >.

### EAU mice showed perivascular inflammatory cell infiltration and subsequent retinal vascular transcytosis

**Fig. 2:**
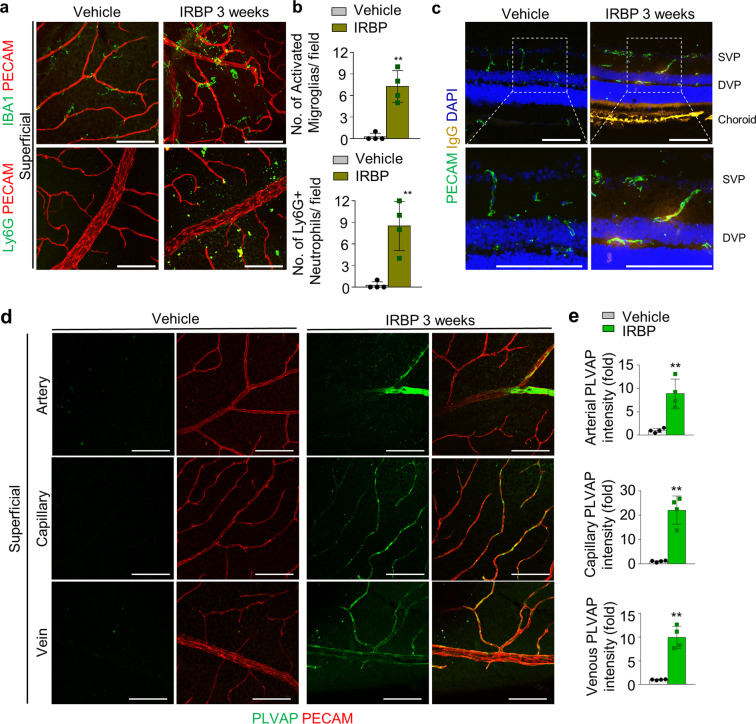
Interphotoreceptor retinoid-binding protein (IRBP)-induced retinal vasculitis is characterized by perivascular inflammatory cell infiltration and vascular leakage in the retina. **a** Mice with experimental autoimmune uveitis (EAU) showed activation of microglia and infiltration of Ly6G+ neutrophils around the retinal vessel. **b** Quantification of the number of activated microglia and Ly6G+ neutrophils (*n* = 4 retinas per group). **c** Cross-section images of the retinas of mice with EAU showing extravasated IgG. The outlined areas are magnified below. DVP: deep vascular plexus; SVP: superficial vascular plexus. **d** Immunostaining of plasmalemma vesicle-associated protein (PLVAP) in whole-mounted retinas showing increased vascular PLVAP expression in mice with EAU 3 weeks after IRBP immunization. **e** Quantification of PLVAP intensity (*n* = 4 retinas per group) according to vessel type. The data are presented as the mean ± SD. Scale bars, 100 μm (**a**, **c**, **d**). *P < 0.05, **P < 0.01.

Because human noninfectious uveitis is characterized by retinal vasculitis, as evidenced by perivascular leakage^29^, we examined the activity and recruitment of inflammatory cells and the level of transcytosis through endothelial cells (Fig. 2). After immunization with IRBP and infiltration of activated antigen-specific T cells into the retina, subsequent activation of local microglia and neutrophil recruitment to perivascular areas were observed (Fig. 2a, b). Notably, mice with EAU showed extravasation of IgG at pericapillary areas (Fig. 2c) and an increased level of transcytosis, as evidenced by elevation of plasmalemma vesicle-associated protein (PLVAP) immunostaining throughout the retinal capillaries and some segments of the arteries and veins (Fig. 2d, e).

**Fig. 3:**
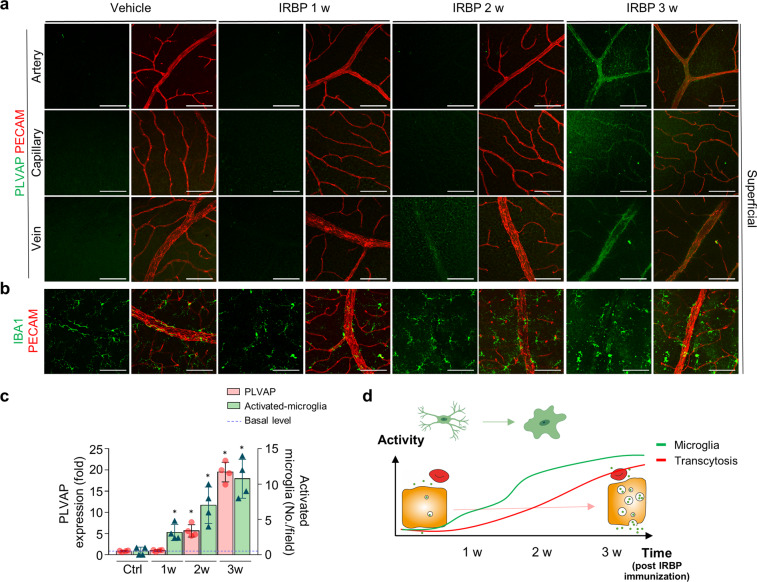
Activation of perivascular microglia precedes increased transcytosis in retinal vessels in interphotoreceptor retinoid-binding protein (IRBP)-induced retinal vasculitis. **a**, **b** Serial images of whole-mounted retinas from mice with experimental autoimmune uveitis (EAU) showing activation of perivascular microglia (**b**) and a subsequent increase in plasmalemma vesicle-associated protein (PLVAP) expression in the retinal vessels (**a**) after IRBP immunization. **c** Quantification of microglial activation and venous PLVAP expression. **d** Diagram depicting the temporal changes in PLVAP expression and microglial activation. Scale bars, 100 μm (**a**, **b**). *P < 0.05.

To gain insights into the temporal relationship between inflammatory cell infiltration and transcytosis induction, activation of local microglia, infiltration of neutrophils, and induction of transcytosis were serially evaluated every week until 3 weeks after IRBP immunization (Fig. 3, Supplementary Figs. 1 and 2). Unexpectedly, following IRBP immunization, microglial activation and neutrophil infiltration, which started after 1 week and preceded PLVAP elevation, gradually increased after 2 weeks (Fig. 3a, b, Supplementary Figs. 1 and 2, Video 1 and 2). These results indicate that inflammatory cell activation might precede and play a role in the increase in vascular permeability via transcytosis induction (Fig. 3c, d).

**Fig. 4:**
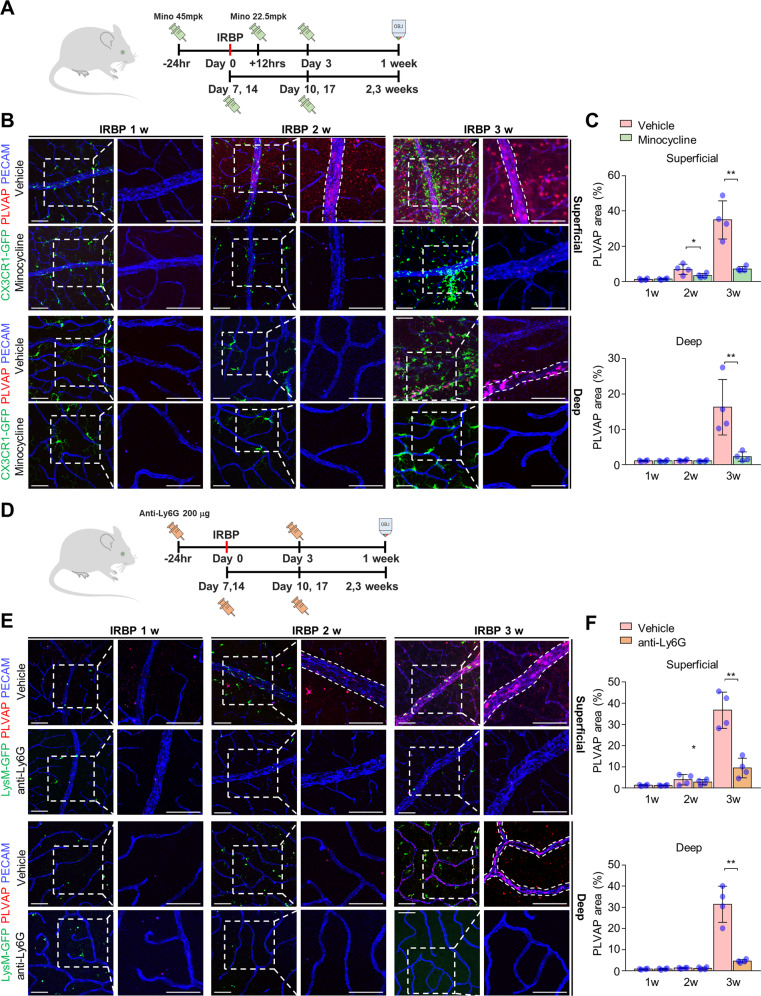
Immune cell suppression ameliorates transcytosis in the retinas of mice with interphotoreceptor retinoid-binding protein (IRBP)-immunized. **A** Schematic diagram of the experimental schedule for the minocycline-induced microglial suppression model. **B** Serial images of whole-mounted retinas from minocycline-injected *Cx3cr1-GFP* mice with experimental autoimmune uveitis (EAU) showing decreased microglial activity and decreased PLVAP expression. **C** Quantification of microglial activation and PLVAP expression. **D** Schematic diagram of the experimental schedule for the anti-Ly6G antibody-induced neutrophil suppression model. **E** Serial images of whole-mounted retinas from anti-Ly6G antibody-injected *LysM-GFP* mice with EAU showing decreased neutrophil infiltration and induction of PLVAP expression. **F** Quantification of neutrophil infiltration and PLVAP expression. The insets are magnified images of PECAM and PLVAP in the outlined areas. Scale bars, 100 μm (**B**, **E**). *P < 0.05, **P < 0.01.

To explore the causal relationships between inflammatory cell activation and transcytosis induction, we designed a rescue experiment in which neutrophil infiltration and microglial activation were blocked in mice with EAU (Fig. 4, Supplementary Fig. 3). First, we suppressed microglial in *Cx3cr1-GFP* mice, which are commonly used as microglial reporter mice, with EAU using minocycline (Fig. 4A)^23^. Interestingly, minocycline-induced microglial inactivation significantly attenuated PLVAP induction in the superficial and deep retinal vessels in the retinas of mice with EAU 2 and 3 weeks after IRBP immunization (Fig. 4B, C). Second, to block neutrophil infiltration in the retinas of mice with EAU, we inhibited neutrophil inhibition in *LysM-GFP* mice (neutrophil-reporter mice) with an anti Ly6G-antibody (Fig. 4D)^30,31^. At 3 weeks after IRBP injection, anti-Ly6G antibody-induced neutrophil inhibition significantly inhibited vascular transcytosis in mice with EAU, which is consistent with the results observed in minocycline-injected mice (Fig. 4E, F). Overall, these results suggest that neutrophils and microglia are involved in regulating vascular permeability and interact under uveitic conditions.

### Intravital and angiographic analysis of mice with EAU

**Fig. 5:**
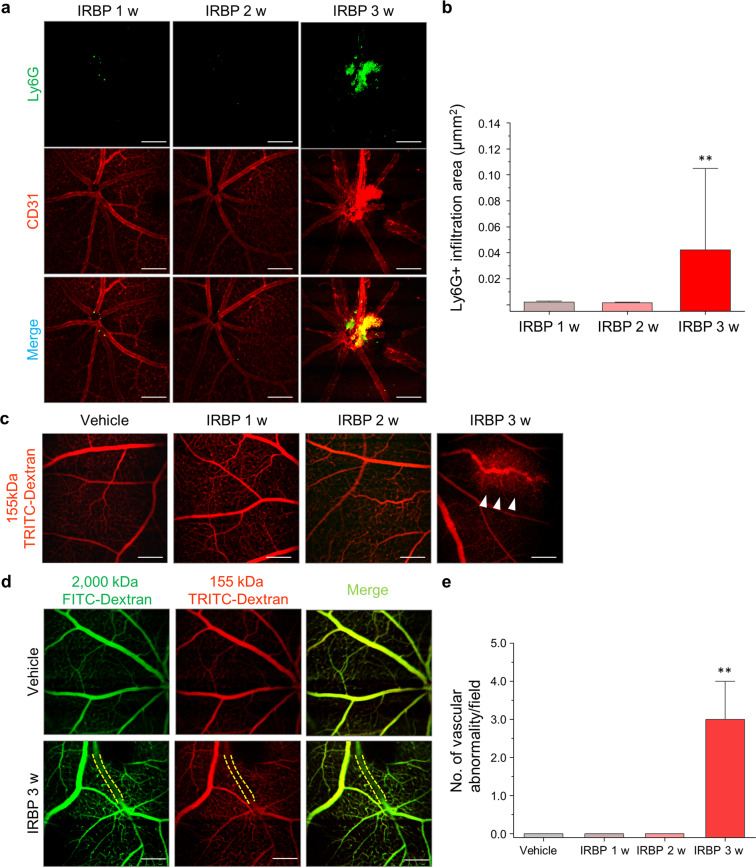
Intravital images of the retinas of interphotoreceptor retinoid-binding protein (IRBP)-immunized mice. **a** Representative intravital images of mice with experimental autoimmune uveitis (EAU). Mice with EAU showed sequestration of Ly6G+ neutrophils. **b** Quantification of Ly6G+ cell infiltration (*n* = 6 retinas per group). **c** Representative intravital images of mice with EAU showing vascular leakage 3 weeks after IRBP immunization. The arrowhead indicates an area of vascular leakage. **d** Representative intravital images of the retinas of mice with EAU that were administered different dextran-conjugated tracers. **e** Quantification of the number of vascular abnormalities (vascular occlusion and leakage, optic nerve leakage; *n* = 6 for controls and *n* = 8 for mice with EAU). Scale bars, 100 μm. **P < 0.01.

We further studied intravital changes in mice with EAU by performing in vivo retinal fluorescence imaging with a custom-built confocal laser-scanning microscope^19,32^. Consistent with our histologic data, Ly6G+ neutrophil infiltration and vascular sequestration were evident 3 weeks after IRBP immunization (Fig. 5a, b). To evaluate functional vascular leakage in the retinas of mice with EAU in vivo, we systemically injected FITC- and TRITC-conjugated dextran (molecular weight: 4400–2,000,000 Daltons) and obtained real-time intravital confocal images of the retina. Vascular leakage was difficult to detect before 2 weeks. However, partial obstruction and some leakage of the retinal vessels (particularly in the optic nerve head) were observed in mice with EAU 3 weeks after IRBP immunization (Fig. 5c–e).

**Fig. 6:**
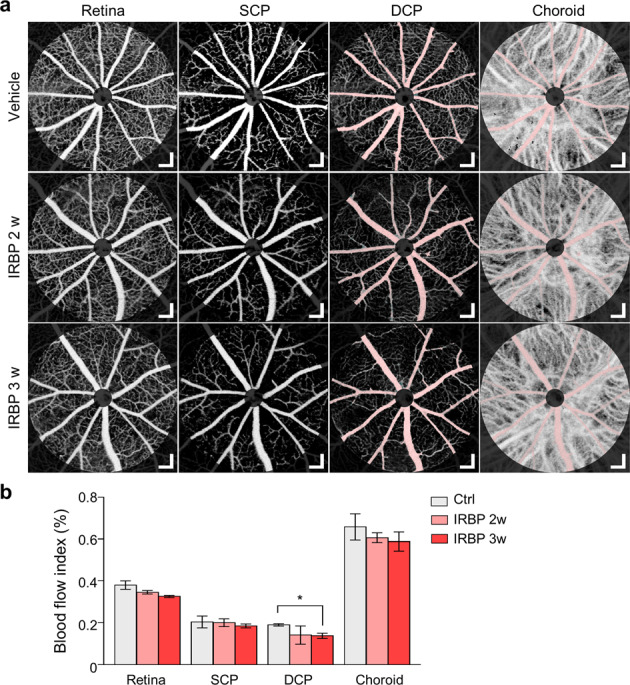
Optical coherence tomography angiography (OCTA) of the retinas of interphotoreceptor retinoid-binding protein (IRBP)-immunized mice. **a** Representative OCTA images of the vascular layers of mice with experimental autoimmune uveitis (EAU). The pinkish area is a shadow mask from a large vessel in the retina, and areas near the optic nerve head were excluded from the ROI because of lens reflection artifacts. DCP: deep capillary plexus; SCP: superficial capillary plexus. The retina includes the total retinal vascular plexus, including the SCP and DCP. **b** Quantification of the blood flow index (BFI) according to vascular layers. Scale bars, 100 μm (**a**). *P < 0.05 compared to the vehicle control; Dunnett’s post-hoc test.

To examine whether mice with EAU exhibit vascular changes in the deeper layer of the retina, we employed custom OCTA for analysis of the retinal vessels according to layer. Notably, after weekly examination of mice with EAU for 3 weeks, we found that the blood flow index (BFI) of the deep capillary layer was significantly decreased in mice with EAU compared to vehicle controls (Fig. 6). Along with the deep capillary layer, the other layers of the retina (the superficial capillary layer and choriocapillaris) showed a trend toward a decrease in BFI in mice with EAU compared to controls, although the differences were not statistically significant (Fig. 6b). These data demonstrate that IRBP immunization induces a decrease in vascular flow, which can be sensitively detected in the deep capillary layer of the retina, where a higher proportion of capillaries and venules are located.

### Functional analysis of mice with EAU

**Fig. 7:**
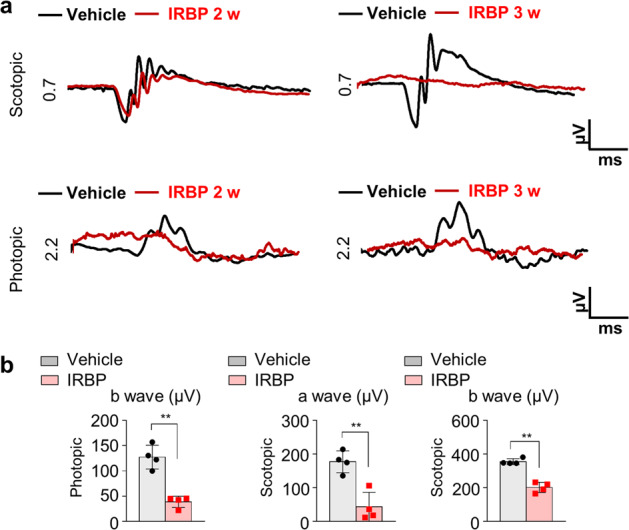
Electroretinogram (ERG) of mice with experimental autoimmune uveitis (EAU). **a** Representative ERG data showing that the amplitudes of both photopic and scotopic responses were decreased in mice with EAU. **b** Quantification of the amplitude of photopic b waves and scotopic a waves 3 weeks after IRBP immunization (*n* = 4 retinas per group). **P < 0.01 compared to the vehicle control.

**Fig. 8:**
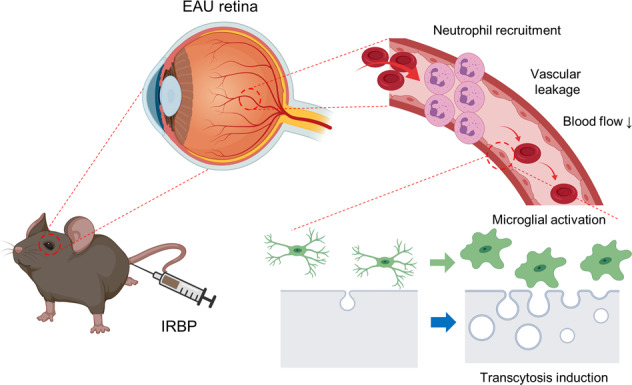
Schematic illustration showing the process of retinal vascular pathology in interphotoreceptor retinoid-binding protein (IRBP)-induced experimental autoimmune uveitis. Images were created using BioRender. Video 1. 3D renderings of microglia in the control retina. Video 2. 3D renderings of microglia in the retinas of mice with experimental autoimmune uveitis mouse 3 weeks after interphotoreceptor retinoid-binding protein administration.

Because we histologically observed extensive photoreceptor loss and retinal folding, that which significantly affect visual function, in mice with EAU, we performed electroretinogram (ERG) to evaluate visual function. Some scotopic responses were intact 2 weeks after IRBP immunization; however, normal photopic and scotopic responses were hardly detected at 3 weeks (Fig. 7a, b). These ERG findings indicate that cascades of reactions, such as neutrophil recruitment, decreased vascular flow, microglial activation, and transcytosis induction, might eventually contribute to visual impairment in mice with EAU (Fig. 8).

## Discussion

In this study, we identified the macro- and microstructural characteristics of retinal vessels in mice with EAU, possibly elucidating the pathophysiology of vasculitis in patients with noninfectious uveitis. The multimodal experimental analysis performed herein, which involved immunostaining, in vivo intravital fundus imaging, live confocal imaging, OCT, OCTA, and ERG revealed the vascular and perivascular changes that underlie EAU. By using these techniques, we showed that neutrophil infiltration and perivascular microglial activation preceded transcytosis induction, which was followed by a decrease in vascular flow, in mice with EAU. Inflammatory cell activation in the perivascular area might precede and play a major role in triggering vascular leakage via transcytosis induction in retinal vasculitis.

CNS tissues such as the brain and retina are highly metabolic and require high blood flow; however, their vessels have low permeability and are highly controlled^33^. Therefore, unlike in peripheral vessels, substances in the blood cannot easily be transported across the BBB/BRB into neural tissue. This transport is meticulously regulated and restricted by endothelial tight junctions to prevent paracellular transport and a low rate of transcytosis to prevent transcellular transport^34^. Transcytosis is the transport of substances through vesicle formation from plasma membrane rafts on the luminal surface, vesicle transport across the endothelial cytoplasm, and vesicle release after docking to the abluminal surface, or vice versa^35^. Changes in retinal transcytosis regulation under hypertensive and diabetic conditions were previously studied by our group^14,17,23^; nonetheless, inflammation-induced transcytosis induction has not been explored.

In this study, we demonstrated that vascular hyperpermeability via transcytosis induction was closely associated with inflammatory cell activation in the perivascular area. Neutrophil infiltration and microglial activation were dominant in large vessels in the optic nerve and perivenous and relevant capillary areas. This was comparable to a previous report indicating that acute-phase inflammation (days 10 to 28) occurred in the optic disc and venules^36^. Although the proportion of neutrophils is relatively lower (10% of infiltrating cells) than that of monocytes (40% of infiltrating cells), infiltrating neutrophils are significantly related to retinal damage^37^. The major contributing cells remain elusive; however, rodents with EAU show significant elevation of vascular endothelial growth factor (VEGF) and transforming growth factor-β (TGFβ) levels in the inner retina, possibly due to production of these factors by mononuclear phagocytes (activated microglia and monocyte-derived macrophages) and neutrophils^38^. As VEGF and TGFβ are well known to disrupt the BRB by increasing transcytosis^39^, these environmental changes might increase transcytosis and induce vascular leakage. Nevertheless, as we do not have direct evidence regarding these growth factors, caution should be taken in interpreting the sequence of events that lead to transcytosis induction in EAU.

Our data showed that microglial activation and subsequent recruitment of leukocytes, as well as vascular obstruction, occurred 2 weeks after immunization. Microglial activation induces leukocyte recruitment by increasing the adhesion of leukocytes to the vessel wall in mice with EAU^40^. Therefore, we suspect that microglial activation might lead to focal sequestration of leukocytes, eventually yielding a decrease in blood flow and vascular leakage via transcytosis induction. This hypothesis was partially supported by our data showing that blocking neutrophil infiltration or microglial activation rescued PLVAP induction in EAU. However, the involvement of neutrophils and microglia in regulating endothelial transcytosis may be different depending on the stage of inflammation, as neutrophil inhibition in the early phase of EAU did not reduce PLVAP levels. Vascular abnormalities in human noninfectious uveitis include vascular occlusion, leakage, edema, local ischemia, and neovascularization^41^. Thus, OCTA can be utilized to accurately detect anatomical and functional vascular changes in the deep retinal layers and provide supportive data that may reflect the inflammatory status.

Notably, the decrease in blood flow in mice with EAU, which was detected by BFI with OCTA, was more evident in the deep vascular layer than in the other vascular layers. As the retinal deep vascular layer comprises a large proportion of small vessels, such as capillaries and venules, in which blood flow is slow, the chance that inflammatory cells will contact the endothelium is high, and this contact may sensitively reflect inflammatory changes in the vessels^14,42^. Currently, there is no generally accepted cutoff value for deep retinal blood flow reduction, which can cause visual impairment and ischemic damage to photoreceptors and the outer retina. Our data revealed the range of pathophysiological changes in response to IRBP immunization that resulted in functional visual impairment, which began at ~2 weeks postimmunization and peaked at 3 weeks and was indicated by a significant decrease in b- and a-wave amplitudes in both scotopic and photopic responses. These findings were comparable to those of a previous study^39^. However, clinically, there is a temporal gap between blood flow changes and related anatomical and functional changes in the retina, such as in patients with ischemic retinopathies (e.g., retinal vein occlusion, diabetic retinopathy)^40,41^. Therefore, caution should be exercised when interpreting these pathophysiological changes, and further comprehensive studies are needed to clarify this issue.

There are some limitations to our study. First, the EAU model induced by IRBP immunization does not recapitulate chronic recurrent uveitis. Although there are reports that low-grade inflammation persists until 60 days postimmunization^36^, uveitis gradually abates after 3 weeks postimmunization^43–45^. Given that chronicity and recurrence are significant issues in uveitis treatment, further studies using a uveitis model that incorporates these features might aid our understanding of this pathophysiology. Second, our study lacked a comprehensive immunologic analysis of the association between specific immune cell types and retinal vascular changes. As immunologic evaluation and lineage tracing of immune cells were beyond the scope of our study, the relationship between retinal vessels and immune cells other than microglia and neutrophils (e.g., macrophages) in EAU remains an important question to be explored in future studies. Third, we did not investigate other uveitogenic antigens, such as s-Ag, the RPE65 protein, and the IRBP protein. Therefore, caution should be exercised when applying our findings to other uveitis models^46^. Fourth, our data revealed a phenotypic discrepancy between the extent of extravasation and the blood flow index. It is natural to show a more prominent leakage response in the choriocapillaris because it is composed of a greater number of endothelial fenestrations and exhibits higher levels of PLVAP than retinal endothelial cells, which lack fenestrations and PLVAP expression^16,45^. The choroid accounts for approximately 85% of the retinal blood supply and supplies oxygen and nutrients to the retinal pigment epithelium and photoreceptors of the outer retina^46^. Because of its inevitable role in the retinal supply, the choroid is richly innervated by the autonomic nervous system and trigeminal sensory nerve fibers that regulate blood flow of the choroids. Although leakage may be significantly increased in the choriocapillaris in our model, regulatory mechanisms that alter choroidal perfusion pressure may compensate for this fluid loss, thereby maintaining the homeostasis of blood flow. As the retinal vessels are devoid of these autonomic innervations, these differences in vascular innervation may lead to phenotypic differences in the retina and choroid^47^.

In summary, we showed the retinal vascular characteristics of mice with EAU using multimodal imaging. We showed that inflammatory cell activation and subsequent transcytosis induction in endothelial cells might be major pathogenic factors of vascular leakage in uveitis. Our data provide information about vascular and perivascular changes in EAU and may provide new insights into the pathophysiology of retinal vasculitis in noninfectious uveitis. We suggest that the IRBP immunization-induced EAU model is an excellent experimental model for studying inflammation-induced BRB breakdown in retinal vasculitis.

## Supplementary information

Supplementary figures

Video 1

Video 2
