# Supplementary material for: Multimodal evaluation of an interphotoreceptor retinoid-binding protein-induced mouse model of experimental autoimmune uveitis
Source: Exp Mol Med. 2022 Mar 9;54(3):252–62. doi: 10.1038/s12276-022-00733-z (PMC8979956; doi:10.1038/s12276-022-00733-z)
Supplement: Supplementary file 1 — Supplementary figures [file 12276_2022_733_MOESM1_ESM.pdf]

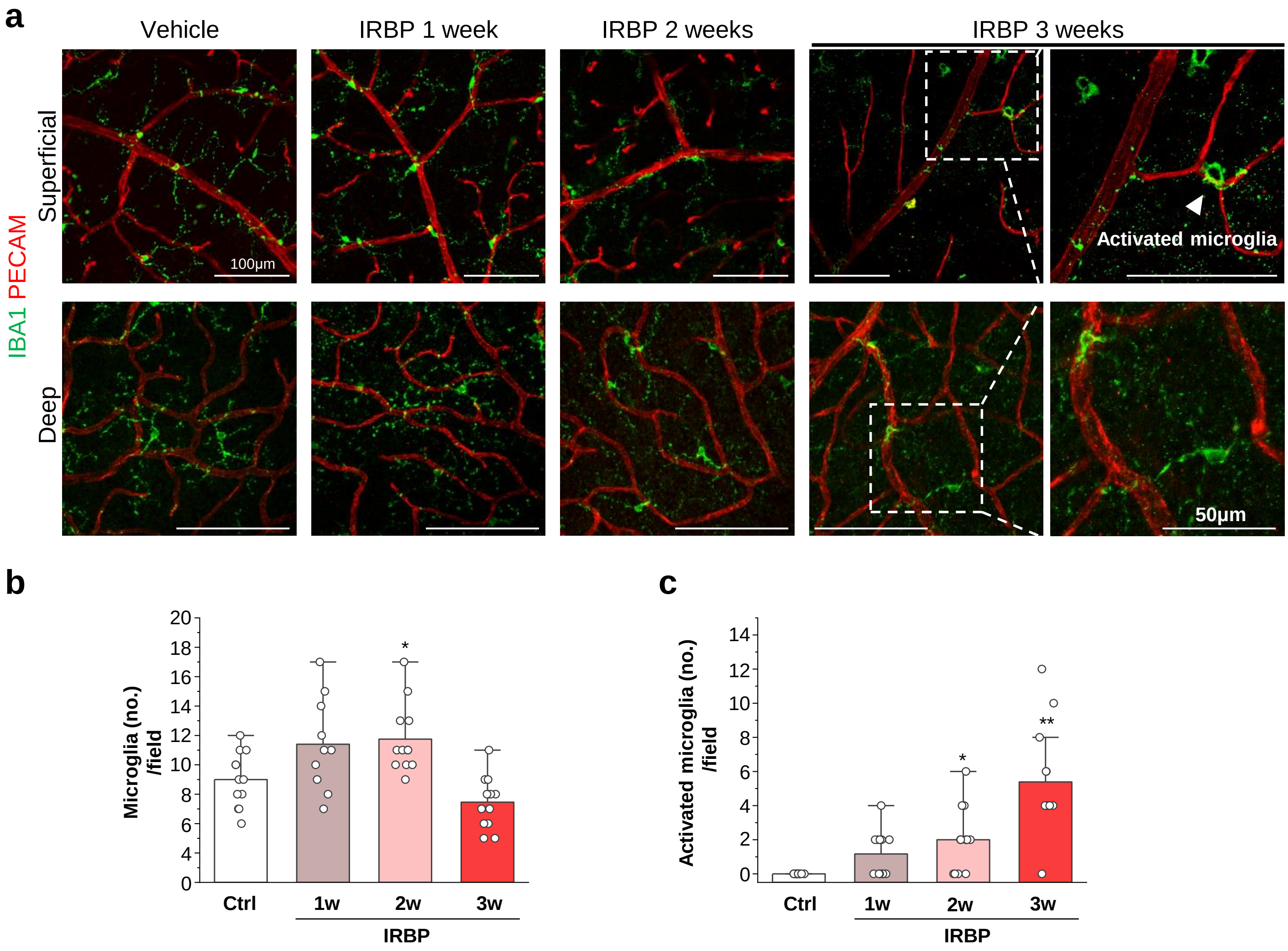

Supplementary Fig. 1. Serial images of whole-mounted retina showing changes of the microglia activation in the superficial and deep vascular layer after interphotoreceptor retinoid-binding protein immunization. (a) Confocal images of the whole-mounted retina. (**b**) Quantifications (n = 6–12 retinas per group) of the number of the total microglia and (**c**) activated microglia in superficial and deep vascular layer by analyzing 3D rendering images with high-resolution Z stacks (supplementary video 1 and 2). Scale bars, 100 μm. $^{*}P < 0.05$ ** $P < 0.01$ compared to controls.

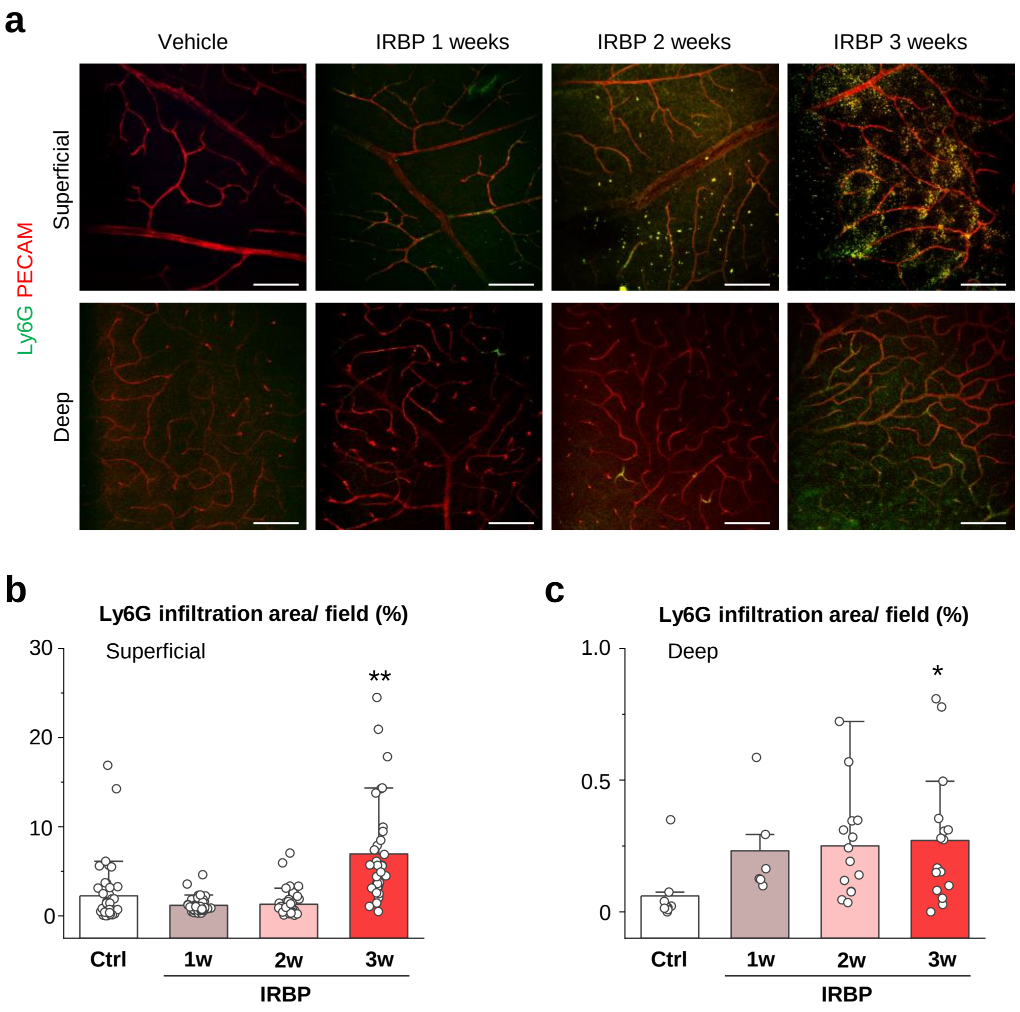

Supplementary Fig. 2. Serial images of whole-mounted retina showing changes of the neutrophil infiltration in the superficial and deep vascular layer after interphotoreceptor retinoid-binding protein immunization. (a) Confocal images of the whole-mounted retina and **(b, c)** its quantifications in each layers (n= above 10 retinas per group). Scale bars, 100 μm. $^{*}P < 0.05$ $^{**}P$ <0.01 compared to controls.

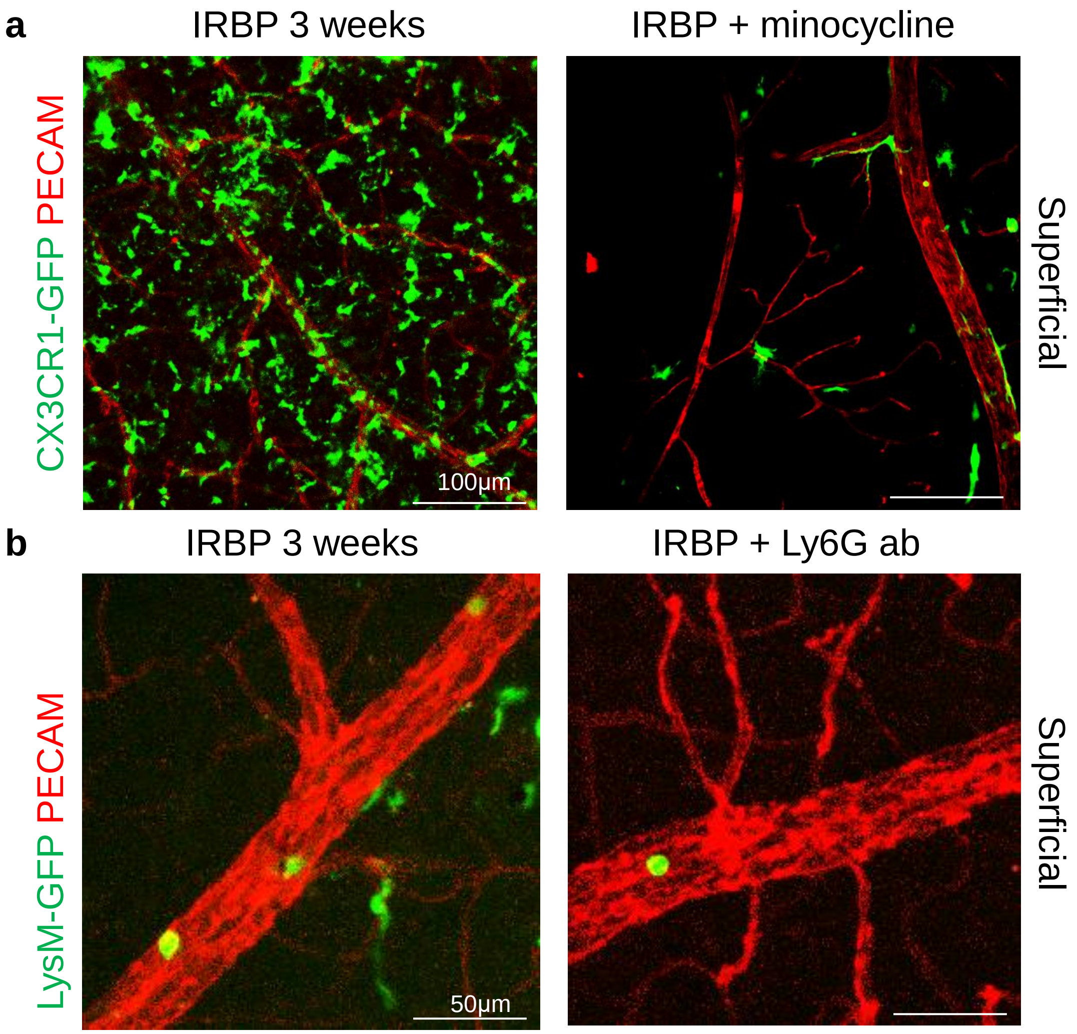

Supplementary Fig. 3. Validation of minocycline-induced microglial suppression and Ly6G-induced inhibition of neutrophil infiltration. (a) Confocal images of the whole-mounted retina of the experimental autoimmune uveitis (EAU) mouse with and without minocycline injection. **(b)** Confocal images of the whole-mounted retina of the EAU mouse with and without Ly6G injection. Scale bars, 100 μm.
